# Self-Assembled Films of Dendrimers and Metallophthalocyanines as FET-Based Glucose Biosensors

**DOI:** 10.3390/s111009442

**Published:** 2011-10-03

**Authors:** Nirton C.S. Vieira, Alessandra Figueiredo, Alvaro A.A. de Queiroz, Valtencir Zucolotto, Francisco E.G. Guimarães

**Affiliations:** 1 Instituto de Física de São Carlos, Universidade de São Paulo, CP 369, São Carlos, SP 13560-970, Brazil; E-Mails: afigueiredo17@gmail.com (A.F.); zuco@ifsc.usp.br (V.Z.); guimaraes@ifsc.usp.br (F.E.G.G); 2 Instituto de Ciências Exatas, Universidade Federal de Itajubá, CP 50, Itajubá, MG 37500-903, Brazil; E-Mail: alencar@unifei.edu.br

**Keywords:** glucose biosensor, layer by layer, field effect transistor, enzyme immobilization

## Abstract

Separative extended gate field effect transistor (SEGFET) type devices have been used as an ion sensor or biosensor as an alternative to traditional ion sensitive field effect transistors (ISFETs) due to their robustness, ease of fabrication, low cost and possibility of FET isolation from the chemical environment. The layer-by-layer technique allows the combination of different materials with suitable properties for enzyme immobilization on simple platforms such as the extended gate of SEGFET devices enabling the fabrication of biosensors. Here, glucose biosensors based on dendrimers and metallophthalocyanines (MPcs) in the form of layer-by-layer (LbL) films, assembled on indium tin oxide (ITO) as separative extended gate material, has been produced. NH_3_^+^ groups in the dendrimer allow electrostatic interactions or covalent bonds with the enzyme (glucose oxidase). Relevant parameters such as optimum pH, buffer concentration and presence of serum bovine albumin (BSA) in the immobilization process were analyzed. The relationship between the output voltage and glucose concentration shows that upon detection of a specific analyte, the sub-products of the enzymatic reaction change the pH locally, affecting the output signal of the FET transducer. In addition, dendritic layers offer a nanoporous environment, which may be permeable to H^+^ ions, improving the sensibility as modified electrodes for glucose biosensing.

## 1. Introduction

The field effect transistor (FET) concept, combined with the high specificity of enzymes, has led to the development of a series of sensing devices to measure analytes of clinical and environmental interest [1,2]. Particularly, ion sensitive field effect transistors (ISFETs) were the first and the most used type of sensor due to the possibility of large scale manufacture [3]. On the other hand, in ISFETs, the FET is in direct contact with the solution, which can hinder the measurement process and due to its micro dimensions, the immobilization of biomolecules is not trivial. Alternatively, the separative extended gate FET (SEGFET) technology [4,5] has been particularly useful for this task as the FET can be isolated from solution and the measurement system is simpler when compared with the traditional ISFET. In addition, there is no need to construct the MOSFET, so it can be used again in new measurements representing a simple and effective route for preparing FET-based biosensors.

Glucose biosensors are the most studied ones, since high glucose levels in human blood is not desirable, being an indicative of diabetes mellitus, a metabolic disorder that results from defects in the functioning of the pancreas. The measuring principle of FET-based glucose biosensor proposed in this work is based on pH deviation, caused by the following reaction [6]:

(1) $$\beta \text{-D-glucose}+O_{2}\overset{\mathrm{GOx}}{\to }\text{gluconate}+H^{+}+H_{2}O_{2}$$

A FET-based glucose biosensor detects the variation in H^+^ ion concentration resulting from the oxidation of glucose molecules by glucose oxidase (GOx) immobilized on a gate insulator. Silane reagents are necessary to modify these gate insulators, since originally they have no reactive groups for enzyme attachment [4,7].

The electrostatic layer-by-layer (LbL) technique represents a simple and low cost way to combine and control some materials at the molecular level, including even biomolecules [8,9]. Organic, inorganic and some biomolecules, with oppositely reactive groups, can be combined resulting in composites with distinct and unique properties when self-assembled into substrates of different kinds and sizes. In this way suitable systems are formed on the last layer for enzyme immobilization and these self-assembled platforms can be easily implemented as extended gates to be applied in FET-based biosensors.

Recently we have introduced a SEGFET pH sensor based on LbL films of poly(propylene imine) dendrimer (PPI) and nickel tetrasulphonated phthalocyanine (NiTsPc) self-assembled on indium tin oxide (ITO) or gold (Au) as separative extended gate [10]. This system exhibited good sensitivity over a pH range from 4 to 10. In LbL assembly, PPI presents a high density of functional NH_3_^+^ peripheral groups, an ideal system for covalently binding enzymes. In addition, dendrimer layers offer a porous environment, which allows the ionic diffusion through the multilayers down to the ITO, improving the sensibility of the SEGFET pH sensor.

In the present paper, we describe the fabrication and operation of a FET-based glucose biosensor using glucose oxidase immobilized onto multilayered LbL films of cationic PPI and anionic NiTsPc. Relevant parameters of the films’ preparation and enzyme immobilization were examined and optimized previously [10]. The results showed that the presence of bovine serum albumin (BSA) increases the ENFET signal, and that parameters such as pH and ionic strength are also relevant. The relationship between the output voltage and glucose concentration demonstrates that PPI/NiTsPc-GOx FET-based biosensor is able to detect glucose.

## 2. Experimental Section

### 2.1. Chemicals

Glucose oxidase (EC 1.1.3.4 type VII) from *Aspergillus niger* having 100 units/mg of activity, serum bovine albumin (BSA), NiTsPc and glutaraldehyde (GA) were purchased from Sigma Aldrich and used without purification. PPI dendrimer (generation 3) was synthesized by a divergent route from an ethylenediamine (EDA) core as described elsewhere [10]. All other reagents were of analytical grade and used as received. ITO coated glasses (160 nm) were purchased from Delta Technologies and were cleaned by immersion in a mixture of HNO_3_-HCl-H_2_O (1:3:20) for 10 min, followed by washing in Milli-Q water (18.3 MΩ·cm).

### 2.2. PPI/NiTsPc Growth and GOx Immobilization

PPI/NiTsPc multilayers were assembled onto ITO substrates by immersing the substrates in polycationic PPI solution (1 mg·mL^−1^) for 5 min and anionic NiTsPc solution (0.5 mg·mL^−1^) for 3 min. After each immersion, the pre-coated ITO film was washed with Milli-Q water for 10 seconds and dried under a nitrogen flow. Five bilayers of PPI/NiTsPc were achieved based on our previous work upon repetition of the cycle described above. More details of the PPI/NiTsPc growth and characterization can be found elsewhere [10]. GOx was cross-linked on the five PPI/NiTsPc bilayers with a last layer composed of PPI having NH_3_^+^ terminated groups by dropping 10 μL of a mixture of GOx, BSA and GA. For this, 100 μL of glutaraldehyde (2.5% in water) was mixed with 240 μL of a mixture containing 20 mg·mL^−1^ of BSA and 50 mg·mL^−1^ of GOx according to the previously described methodology [11]. Another membrane without BSA was also tested.

### 2.3. FET-Based Biosensor Measurement System

**Figure 1.**
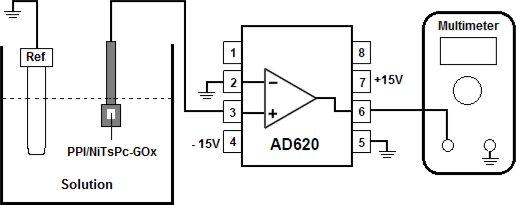
Schematic illustration of the FET-based biosensor measurement system showing the connection diagram of the instrumentation amplifier AD620 used as unity gain buffer.

The FET-based biosensor device was composed of a biochemically sensitive membrane formed by PPI/NiTsPc-GOx as enzyme separative extended gate, connected to a commercial AD620 amplifier used here as unit gain buffer. The biomembrane was immersed in a phosphate buffer solution (10 mM, pH 7.5) and glucose aliquots were then added in the measurement cell to determine the glucose sensing characteristics. For the measurements of the time dependence on the output voltage we used a Ag/AgCl/Sat-KCl reference electrode to support a constant voltage. The AD620 output voltage was recorded using a Keithley 195A multimeter. Figure 1 illustrates the FET-based biosensor structure and the measurement system, showing the connection diagram of the instrumentation amplifier AD620.

## 3. Results and Discussion

### 3.1. Glucose Response

**Figure 2.**
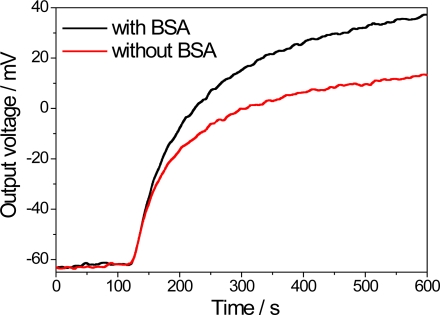
Response of PPI/NiTsPc-GOx FET-based biosensor with (black line) and without (red line) the presence of BSA in the process of enzyme immobilization to detect 0.5 mM of glucose. Measurement conditions: buffer solution 10 mM, pH 7.5.

The enzyme immobilization process is an important step in biosensor fabrication since the enzyme cannot be lixiviate from the support. GA is an extensively used cross-linking agent in enzyme immobilization processes in combination with BSA. Figure 2 displays the typical response of the PPI/NiTsPc-GOx FET-based biosensor and the influence of the BSA on the output signal. A response time—defined as the time necessary to reach 90% of the steady-state response [12]—of about 7 min can be estimated. This time appears to be slightly lower for the biosensor without BSA due to the faster ionic diffusion, as the presence of BSA may make the H^+^ diffusion difficult. On the other hand, the presence of BSA allows greater exposure of the enzyme active sites, since they are not used in the cross-linking process, enhancing the enzyme activity and thus improving the biosensor signal.

**Figure 3.**
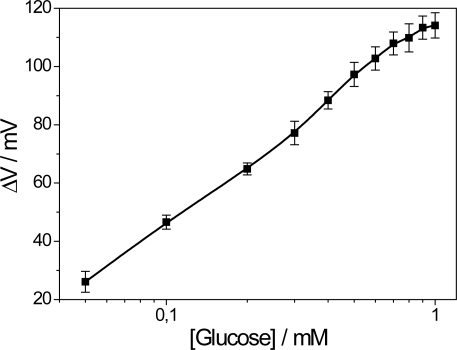
Analytical curve of the PPI/NiTsPc-GOx FET-based biosensor. Measurement conditions: buffer solution 10 mM, pH 7.5

The dependence of the voltage response *versus* glucose concentration (analytical curve) is shown in Figure 3. The linear range is up to 0.4 mM with detection limit of 0.027 mM determined in the linear region of the biosensor calibration curve according to the literature [13].

The greatest obstacle in the development of FET-based glucose biosensors is their dynamic range limitation due to the low oxygen concentration of real blood samples. Although at first a sensitivity of up to 1 mM seems to be out of the range of interest, which is about 4–7 mM for real blood samples, our sensor is able to detect diluted human serum samples as suggested in the literature [14]. Using such a strategy, the sensors proposed here could be used clinically. Nevertheless, extensive efforts have been directed aiming to solve this problem by using inorganic [4,15] or organic molecules [16] as oxidizing agents or yet directly electrolyzing hydrogen peroxide (H_2_O_2_) using a platinum electrode [7,17]. The breakdown of the H_2_O_2_ molecule resulting from the oxidation of glucose molecule produces two protons and one oxygen molecule in a more reactive environment. These sub-products increase the signal and the oxygen concentration in biosensor measuring media, which helps to extend the biosensor’s dynamic range. In fact, metallophthalocyanines may act as artificial enzymes due to their catalytic properties and ionic selectivity, including oxi-reduction of H_2_O_2_ [18,19]. Contrary to our expectations, in the PPI/NiTsPc-GOx FET-based glucose biosensor, NiTsPc only acts as a counter-ion for LbL growth, since the biosensor response is limited to 1 mM. In addition, the diffusion of H_2_O_2_ molecules through the LbL film is improbable so that just a few molecules interact with the phthalocyanine, hindering the catalytic action of NiTsPc.

### 3.2. Influence of the Buffer Concentration and pH

**Figure 4.**
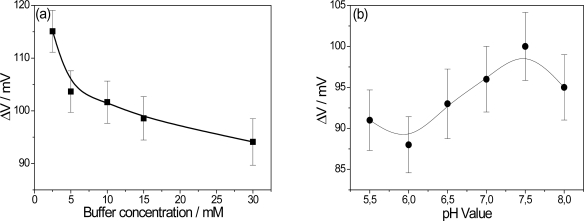
Influence of the buffer concentration **(a)** and effect of pH value on the PPI/NiTsPc-GOx FET-based glucose biosensor response **(b)**.

Upon detection of glucose, the sub-products of the GOx reaction change the pH locally, affecting the electrical properties of the PPI/NiTsPc-GOx FET-based glucose biosensor. It is of importance, then, to study parameters such as pH and buffer concentration, which directly affect the enzyme activity and consequently, the final results. In order to understand the influence of the buffer concentration, studies were made with phosphate buffer over a range of 2.5 mM to 30 mM. It is expected that the pH changes will be more detectable at lower buffer concentrations as a result of minor attenuation of the buffer solution due to ionic strength. The influence of the buffer concentration is shown in Figure 4(a), with the optimum value being achieved of 2.5 mM. However, trying to maintain a certain ionic strength, 10 mM was chosen as the measurement condition.

Aiming to optimize the pH measurement environment, the output voltage was studied within a pH range of 5.5 to 8. The activity of GOx is dependent on the pH value and has an optimum activity in the native form at pH 5.5 according to the manufacturer (Sigma Aldrich). Figure 4(b) shows the effect of pH value on the performance of the PPI/NiTsPc-GOx FET-based glucose biosensor. The biosensor signal increases with the pH value, having its maximum at pH 7.5. This unusual behavior, in contrast with that of a native GOx, can be attributed to the stability of PPI/NiTsPc LbL films as pH sensor. The films are more stable at pH values around 7. The loss of stability at alkaline pH values is a result of charge inversion in the PPI molecule [10]. Our investigations have been carried out under standard conditions, *i.e.*, pH 7.5 and at buffer concentration of 10 mM, close to physiological conditions.

### 3.3. Reuse and Life-Time

**Figure 5.**
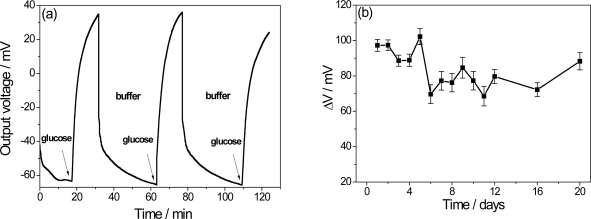
PPI/NiTsPc-GOx FET-based glucose biosensor response after successive immersions in buffer and in buffer solutions containing 0.5 mM of glucose **(a)** and lifetime of the biosensor stored in pH 7.5 buffer solution at 4 °C **(b)**.

The recovery time is an important factor in a biosensor that is expected to perform successive measurements. A delay in the recovery time of the extended electrode is caused by excess H^+^ ions from the enzymatically catalyzed reaction from the previous measurement [7]. In order to investigate this characteristic, Figure 5(a) shows the PPI/NiTsPc-GOx FET-based glucose biosensor response after successive immersions in buffer and in buffer solutions containing 0.5 mM of glucose. A recovery time about 50 min is observed with a small loss of signal, suggesting that the ENFET can be used again. To estimate the lifetime, the biochemically sensitive membranes were stored in buffer solution (pH 7.5) and the measurements were carried out through a few days adding 0.5 mM glucose to the measuring cell. As a result, the Figure 5(b) shows an average of 95 mV in the signal in the early days, with decreases by up to 18.5% after long times, leading to the conclusion that the biosensor can be stored for a period of 20 days without significant loss of sensitivity.

## 4. Conclusions

FET-based glucose biosensors were successfully built, with the advantage of being a low-cost device, in comparison with the usual ISFETs. Multilayered films of PPI and NiTsPc sensitive to pH variations were used as supports for GOx enzyme immobilization in order to obtain an active platform. The PPI/NiTsPc-GOx system showed sensitivity to variations of glucose in a linear range around 0.4 mM with detection limit of 0.027 mM, which is also interesting from a medical point of view as high glucose concentration levels in blood, indicate lack of treatment. The presence of BSA enhances the activity of GOx since fewer molecules of the enzyme are involved in the immobilization process. Unexpectedly, NiTsPc did not present catalytic properties, acting only as a counter-ion for LbL film building. Moreover, the use of dendrimers as support allows an effective enzyme immobilization due to the peripheral functional groups on the periphery of the macromolecule and the possible ionic diffusion through the self-assembled PPI/NiTsPc multilayers.
